# Supplementary material for: A comparative analysis of small RNA sequencing data in tubers of purple potato and its red mutant reveals small RNA regulation in anthocyanin biosynthesis
Source: PeerJ. 2023 May 19;11:e15349. doi: 10.7717/peerj.15349 (PMC10202107; doi:10.7717/peerj.15349)
Supplement: Table S1 [file peerj-11-15349-s001.docx]

**Table S1 Length distributions of small RNAs from six libraries**

| Length | SD140_1 | SD140_2 | SD140_3 | SD92_1 | SD92_2 | SD92_3 |
| --- | --- | --- | --- | --- | --- | --- |
| 10 nt | 0.0358% | 0.0284% | 0.0390% | 0.0281% | 0.0272% | 0.0307% |
| 11 nt | 0.0394% | 0.0289% | 0.0475% | 0.0378% | 0.0346% | 0.0404% |
| 12 nt | 0.0439% | 0.0447% | 0.0703% | 0.0570% | 0.0414% | 0.0536% |
| 13 nt | 0.0497% | 0.0577% | 0.0986% | 0.0709% | 0.0510% | 0.0653% |
| 14 nt | 0.0836% | 0.1132% | 0.1521% | 0.1145% | 0.0776% | 0.1070% |
| 15 nt | 0.1198% | 0.1320% | 0.2084% | 0.1413% | 0.1052% | 0.1392% |
| 16 nt | 0.2211% | 0.1796% | 0.3014% | 0.1705% | 0.1496% | 0.2064% |
| 17 nt | 0.3733% | 0.2674% | 0.4681% | 0.2348% | 0.3083% | 0.4096% |
| 18 nt | 0.7836% | 0.3543% | 0.9146% | 0.2895% | 0.5324% | 1.0070% |
| 19 nt | 2.0913% | 0.7661% | 2.1334% | 0.4598% | 2.0050% | 2.5691% |
| 20 nt | 3.7473% | 1.8190% | 3.1660% | 1.7647% | 4.3557% | 4.2606% |
| 21 nt | 19.3529% | 11.9223% | 15.1469% | 17.9185% | 15.7339% | 19.3325% |
| 22 nt | 14.5641% | 13.5417% | 12.2163% | 25.9919% | 17.3494% | 19.1092% |
| 23 nt | 14.2303% | 17.3603% | 15.3581% | 12.5745% | 12.5428% | 12.3350% |
| 24 nt | 39.9365% | 49.2376% | 42.0624% | 33.4075% | 32.6407% | 35.5183% |
| 25 nt | 1.5175% | 1.7601% | 2.0076% | 1.8810% | 3.2668% | 1.6910% |
| 26 nt | 0.7958% | 0.7631% | 1.2669% | 1.0593% | 2.4341% | 0.8860% |
| 27 nt | 0.6888% | 0.6286% | 1.1862% | 1.0165% | 2.2837% | 0.7896% |
| 28 nt | 0.4613% | 0.3925% | 0.8859% | 0.7208% | 1.7427% | 0.5423% |
| 29 nt | 0.3620% | 0.2913% | 0.8253% | 0.6625% | 1.6676% | 0.4343% |
| 30 nt | 0.2074% | 0.1456% | 0.5498% | 0.4634% | 1.2203% | 0.2371% |
| 31 nt | 0.1055% | 0.0736% | 0.3271% | 0.2902% | 0.6665% | 0.1084% |
| 32 nt | 0.1318% | 0.0596% | 0.3061% | 0.3488% | 0.4344% | 0.0891% |
| 33 nt | 0.0361% | 0.0193% | 0.1533% | 0.1626% | 0.1935% | 0.0243% |
| 34 nt | 0.0103% | 0.0050% | 0.0713% | 0.0761% | 0.0803% | 0.0058% |
| 35 nt | 0.0027% | 0.0015% | 0.0202% | 0.0213% | 0.0222% | 0.0009% |
| 36 nt | 0.0010% | 0.0005% | 0.0066% | 0.0061% | 0.0084% | 0.0007% |
| 37 nt | 0.0004% | 0.0003% | 0.0022% | 0.0052% | 0.0040% | 0.0003% |
| 38 nt | 0.0003% | 0.0003% | 0.0011% | 0.0027% | 0.0025% | 0.0003% |
| 39 nt | 0.0009% | 0.0010% | 0.0009% | 0.0015% | 0.0016% | 0.0007% |
| 40 nt | 0.0003% | 0.0001% | 0.0003% | 0.0005% | 0.0011% | 0.0002% |
| 41 nt | 0.0006% | 0.0005% | 0.0009% | 0.0012% | 0.0020% | 0.0005% |
| 42 nt | 0.0010% | 0.0007% | 0.0012% | 0.0033% | 0.0036% | 0.0008% |
| 43 nt | 0.0033% | 0.0027% | 0.0031% | 0.0090% | 0.0063% | 0.0031% |
| 44 nt | 0.0007% | 0.0004% | 0.0009% | 0.0068% | 0.0037% | 0.0005% |
