# Supplementary material for: A comparative analysis of small RNA sequencing data in tubers of purple potato and its red mutant reveals small RNA regulation in anthocyanin biosynthesis
Source: PeerJ. 2023 May 19;11:e15349. doi: 10.7717/peerj.15349 (PMC10202107; doi:10.7717/peerj.15349)
Supplement: Table S2 [file peerj-11-15349-s002.docx]

**Table S2 Alignment statistics of tags aligned to reference genome**

| Sample name | Total tags | Mapped tags | Percentage (%) |
| --- | --- | --- | --- |
| SD140_1 | 28122156 | 24085295 | 85.65 |
| SD140_2 | 28174058 | 24155100 | 85.74 |
| SD140_3 | 26809871 | 22800366 | 85.04 |
| SD92_1 | 26717934 | 23271438 | 87.10 |
| SD92_2 | 27493348 | 23854883 | 86.77 |
| SD92_3 | 28002462 | 24287294 | 86.73 |
