# Supplementary material for: A comparative analysis of small RNA sequencing data in tubers of purple potato and its red mutant reveals small RNA regulation in anthocyanin biosynthesis
Source: PeerJ. 2023 May 19;11:e15349. doi: 10.7717/peerj.15349 (PMC10202107; doi:10.7717/peerj.15349)
Supplement: Table S3 [file peerj-11-15349-s003.docx]

**Table S3 Classification of small RNAs in six potato libraries**

| Type | SD140_1 | SD140_2 | SD140_3 | SD92_1 | SD92_2 | SD92_3 |
| --- | --- | --- | --- | --- | --- | --- |
| intergenic | 83.2953% | 83.6518% | 82.7323% | 84.0952% | 83.1511% | 83.7353% |
| mature | 1.8416% | 1.6912% | 1.3313% | 2.1800% | 1.8049% | 2.4252% |
| sncRNA | 0.0136% | 0.0110% | 0.0242% | 0.0250% | 0.0559% | 0.0238% |
| snRNA | 0.0555% | 0.0628% | 0.0619% | 0.0724% | 0.0510% | 0.0666% |
| unmap | 14.3003% | 14.2062% | 14.8564% | 12.8277% | 13.1260% | 13.1992% |
| rRNA | 0.4512% | 0.3248% | 0.9190% | 0.7440% | 1.7638% | 0.4955% |
| hairpin | 0.0001% | 0.0001% | 0.0001% | 0.0001% | 0.0002% | 0.0003% |
| snoRNA | 0.0053% | 0.0073% | 0.0074% | 0.0096% | 0.0083% | 0.0084% |
| precursor | 0.0362% | 0.0442% | 0.0653% | 0.0421% | 0.0352% | 0.0450% |
| tRNA | 0.0008% | 0.0005% | 0.0021% | 0.0038% | 0.0036% | 0.0007% |
