# Supplementary figures and images for: A comparative analysis of small RNA sequencing data in tubers of purple potato and its red mutant reveals small RNA regulation in anthocyanin biosynthesis
Source: PeerJ. 2023 May 19;11:e15349. doi: 10.7717/peerj.15349 (PMC10202107; doi:10.7717/peerj.15349)

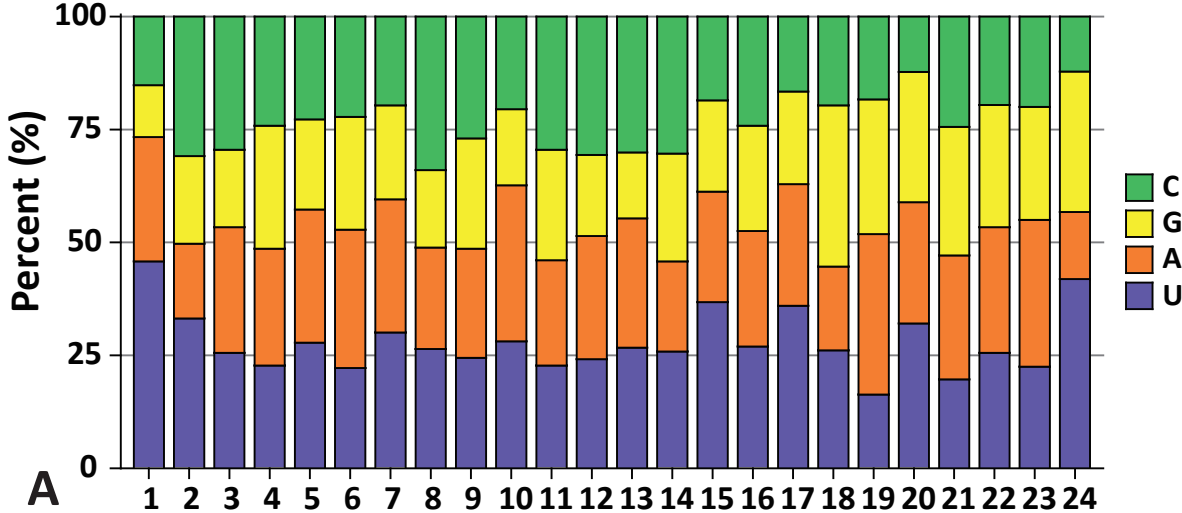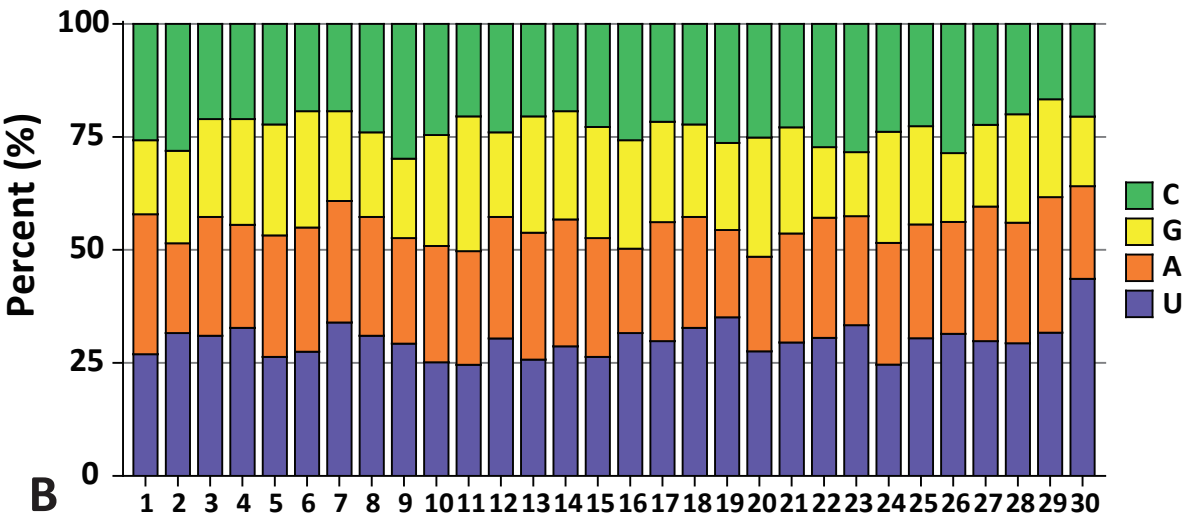

Supplement: Figure S1 — X axis indicates the nucleotide positions of miRNAs. Y axis indicates the percent of 4 nucleotides at each nucleotide position. A, known miRNAs; B, novel miRNAs. [file peerj-11-15349-s011.pdf]
